# Predictive gene lists for breast cancer prognosis: A topographic visualisation study

**DOI:** 10.1186/1755-8794-1-8

**Published:** 2008-04-17

**Authors:** Mingmanas Sivaraksa, David Lowe

**Affiliations:** 1 Neural Computing Research Group, Aston University, Birmingham, UK

## Abstract

**Background:**

The controversy surrounding the non-uniqueness of predictive gene lists (PGL) of small selected subsets of genes from very large potential candidates as available in DNA microarray experiments is now widely acknowledged [1]. Many of these studies have focused on constructing discriminative semi-parametric models and as such are also subject to the issue of random correlations of sparse model selection in high dimensional spaces. In this work we outline a different approach based around an unsupervised patient-specific nonlinear topographic projection in predictive gene lists.

**Methods:**

We construct nonlinear topographic projection maps based on inter-patient gene-list relative dissimilarities. The Neuroscale, the Stochastic Neighbor Embedding(SNE) and the Locally Linear Embedding(LLE) techniques have been used to construct two-dimensional projective visualisation plots of 70 dimensional PGLs per patient, classifiers are also constructed to identify the prognosis indicator of each patient using the resulting projections from those visualisation techniques and investigate whether *a-posteriori *two prognosis groups are separable on the evidence of the gene lists.

A literature-proposed predictive gene list for breast cancer is benchmarked against a separate gene list using the above methods. Generalisation ability is investigated by using the mapping capability of Neuroscale to visualise the follow-up study, but based on the projections derived from the original dataset.

**Results:**

The results indicate that small subsets of patient-specific PGLs have insufficient prognostic dissimilarity to permit a distinction between two prognosis patients. Uncertainty and diversity across multiple gene expressions prevents unambiguous or even confident patient grouping. Comparative projections across different PGLs provide similar results.

**Conclusion:**

The random correlation effect to an arbitrary outcome induced by small subset selection from very high dimensional interrelated gene expression profiles leads to an outcome with associated uncertainty. This continuum and uncertainty precludes any attempts at constructing discriminative classifiers.

However a patient's gene expression profile could possibly be used in treatment planning, based on knowledge of other patients' responses.

We conclude that many of the patients involved in such medical studies are *intrinsically unclassifiable *on the basis of provided PGL evidence. This additional category of 'unclassifiable' should be accommodated within medical decision support systems if serious errors and unnecessary adjuvant therapy are to be avoided.

## Background

Metastasis is crucial in determining the life expectancy of breast cancer patients. Numerous studies have focused on searching for methods to predict the predilection of cancer patients to metastasize. Traditional methods fail to correctly predict the outcome of patients who reach metastasis leading to unnecessary clinical adjuvant therapy, such as chemotherapy. Gene profiling based, for example, on DNA microarray technology, has the potential to be a more reliable method allowing better prediction of patient cancer outcome. However, using lists of many thousands of genes is uninformative and not useful in providing insight for the specialist, nor for discovering the role of specific genes. Feature selection methods have been applied to filter the number of genes which are correlated with outcome to produce a much smaller (typically of the order of a few tens) and more informative 'predictive gene list' (PGL), ideally consisting of the key genes which control the behaviour of the cancer.

Despite the obvious benefits of producing an informative and small PGL, the difficulty is how to perform the feature selection in the absence of good quality functional models of the individual gene pathways. The alternative is to use data-driven data-mining approaches and seek correlations between response and outcome to rank potential genes. To rank the genes requires an appropriate metric. Although feature selection and feature extraction are often unsupervised methods, in the literature in this domain it is more common to be based on a supervised approach based on a specific choice of a nonparametric model linking the gene expressions to the outcomes. For example, using a classification model to infer likely outcome conditioned on expression values allows saliency of individual genes to be obtained, relevant to the classified outcome, e.g. good or poor prognosis patients. This saliency is of course dependent on the chosen model, the pre-specified outcomes, and the specific data used to construct the nonparametric classifier model. It is usually assumed that the data used to construct the classifier is *representative *of the problem so that results obtained are not highly sensitive on the specific choice of data. However, a different choice of model, or different choice of outcome would modify the saliency even if the chosen model was correct. In addition, in problems of such large input dimensionality (5000 or more on microarray chips), and relative sparsity of patient examples (a few hundred is typical), it is statistically plausible to select small subsets which are *randomly *correlated with *any *given desired outcome, *irrespective *of any biological functionality of the gene expression itself. This aspect has already been discussed in [1,2] for example. Therefore the question arises as to whether a specific PGL can be obtained based on clinical datasets, given these concerns over reliability of pattern processing techniques.

Almost all nonlinear studies so far have examined supervised approaches to patient discrimination. A major problem with dealing with such high dimensional data is the lack of reliable approaches to investigate and compare patient-specific gene expression profiles separate to the construction of supervised models. We wish to explore an alternative analysis approach, based on *unsupervised*, nonlinear, topographic (structure-preserving) projection and visualisation methods.

This paper explores several recent nonlinear visualisation models applied to the data introspection of the van't Veer breast cancer study [3]. The approach can be used to *a-posteriori *explore whether there exists likely discriminability between patient groups of good and poor prognosis for example. For comparison with the preferred PGL selected by the van't Veer study, we also select a PGL based on cross-patient consistency rather than correlation with outcome and examine its performance also by these data introspection methods.

### Reviews

We briefly overview some relevant recent works which have explored different classification, discrimination and clustering techniques to represent the separation between two groups of prognosis signature patients. The studies of van't Veer's group [3,4] have suggested that a PGL of 70 specifically selected genes has proven accurate in out-of-sample patient prognosis of metastasis.

However, other studies have concluded that the likelihood there exists a 'best' small-size predictive gene list which can be used to reliably improve the ability of patient-specific prognosis using automated pattern processing techniques is unlikely. In very recent work [5], analysing supervised machine learning approaches across several public domain data sets, it was found that many gene sets are capable of predicting molecular phenotypes accurately. Hence it is not surprising that expression profiles identified using different training datasets selected from a larger cohort, should show little agreement. It was also demonstrated that predicting relapse directly from microarray data using supervised machine learning approaches was not viable.

In other work [6], it was shown that the specific example of the van't Veer PGL selection of 70 genes was no more effective at prognosis than the Nottingham Prognostic Indicator (NPI) or a suitably trained artificial neural network using traditional non-genomic biomarkers. This is not surprising from a systems biology perspective, where we would regard cancer as the result of complex interactions between genetic, biological and environmental influences.

In [1], they also found that the top 70 most correlated genes in the van't Veer study can vary significantly depending on the specific training set of patients used. Different randomly selected 70 gene PGL's were selected and shown to have similar prediction ability. They suggested that there is no unique set of genes that can be assumed to be the best or the only set of genes for prognosis accuracy of breast cancer. A follow-up study [2] also suggested a similar conclusion, that we can not create a definitive classifier from a small subset of genes based on the small patient datasets available. Generally, large patient sample sizes are needed to produce viable and robust prediction outcomes of cancer prognosis.

#### Projective Visualisation

Projective data visualisation is an approach for introspection of large dimensional datasets by extracting useful information and representing it in a more meaningful way that can be more easily interpreted prior to deciding upon subsequent analysis such as constructing classifiers [7]. The approach is very useful for interpreting data by simply observing two or three dimensional projective maps of the original dataspace, where relative positioning of data points reflects some form of structural similarities in the original dataspace. This allows the easy recognition of anomalous data points, outliers, implicit clustering and relative dissimilarity.

In microarray data, the combination of large dimensionality, noise, and sparse patient samples makes it almost impossible to explore and extract useful information contained in the data. Dimensionality reduction techniques are required for visualising microarray data. The dendrogram is one of the traditional approaches to perform microarray data clustering. However it usually produces a suboptimal local clustering solution and is not effective as a spatial visualisation tool to reflect relative dissimilarities. Many other algorithms for reduced dimensionality representation have previously been used to visualise microarray data. For instance, the Self Organising Map (SOM) has been used to investigate yeast [8] and human cancers [9] and [10], the latter in combination with the *k*-means algorithm. Analogously, Principal Component Analysis (PCA) has been used to investigate yeast [11] and to identify tissue-specific expression of human genes [12]. However, both SOM and PCA have significant drawbacks. PCA is a variance-preserving *linear projection*, and this limitation does not lead to a topographic representation [13]. On the other hand, the SOM lacks a sound theoretical underpinning (for example, there is no cost function to optimise, and training parameters must be chosen arbitrarily).

We therefore seek principled approaches to unsupervised data introspection which are nonlinear (since microarray data distributions are unlikely to be distributed on a linear manifold in high dimensional spaces). In this paper we will explore the Neuroscale model [14,15], Local Linear Embeddings (LLE) [16] and Stochastic Neighbor Embeddings (SNE) [17].

## Methods

### The van't Veer Data set

We re-visit the well-known study of van't Veer et.al. [3] in which we focus on 78 sporadic lymph-node negative patients. Of these 78 patients, 34 developed distant metastases within 5 years and 44 remained free of cancer in that period. These are regarded as poor and good-prognosis groups respectively. The interest is whether the information in a gene expression profile alone could be used to perform a patient-specific prognosis separation between those two groups of patients. We will primarily use structure-preserving projective visualisation techniques to investigate this possibility. In the van't Veer study, from an initial set of 24481 human genes synthesised by inkjet microarray technology, about 5000 genes were found to be significantly expressed. They ranked genes by the magnitude of the correlation coefficient and eventually reduced the number of genes to 70, the number of genes which maximised a specific classification model. The centroid-based classifier they constructed could allocate 83% of the patients into the correct prognosis groups with 5 poor prognosis and 8 good-prognosis patients misclassified into the opposite categories.

### An alternative PGL

To illustrate the lack of uniqueness of capability of the van't Veer gene list, which we denote List A in this paper, we compare results on a different gene list, denoted List B, selected on the basis of cross-patient consistency rather than maximising classification accuracy on a specific classification model. Let **x**^*i *^denote the gene expression vector for patient *i *of the van't Veer PGL. **x**_*G*_, where *G *= {1, 2,..., 44} represents a set of expression values across all good prognosis patients, and **x**_*P*_, where *P *= {45, 46,..., 78} represents the set of all poor prognosis patients.

The variance of individual gene expression values across each patient group is estimated by

$$\sigma_{L}^{2}={\langle {\left(x_{i}-{\bar{x}}_{L}\right)}^{2}\rangle }_{i\in L},$$

where *L *= {*G, P*} and the average is taken across all patients. Assume $R_{j}^{L}$ is the rank order of the variance of gene *j *for each patient group. The unique top *T *ranked genes from each group are extracted,

$$\begin{array}{l} L_{G}=\{j|R_{j}^{G}\leq T\}\\ L_{P}=\{j|R_{j}^{P}\leq T\} \end{array}$$

The number of *T *genes is chosen so that List B has a total number of genes equal to 70, the same as List A. Specifically, in this case the 35 lowest non-overlapped variance genes from each patient group were extracted.

List B = {*L*_*G *_∪ *L*_*P*_} - {*L*_*G *_∩ *L*_*P*_}.

This selection criterion emphasises *consistency *of gene expression across patients, rather than explicitly seeking discrimination (see table 25 for list of genes). Examining the details of the two 70-gene subsets, we observe that there are only *five *genes in common between the van't Veer study and this alternative gene list. If List A has superior prognostic value, its projective visualisation and discrimination properties should be better than those of List B, since List A was chosen explicitly to maximise discrimination.

**Table 25:** The alternative gene list

AA553619.RC	AB023216	AB032954	AF065241	AL050065
Contig11065.RC	Contig14706.RC	Contig14882.RC	Contig15031.RC	Contig31839.RC
Contig34302.RC	Contig35229.RC	Contig37063.RC	Contig37262	Contig39090.RC
Contig42162.RC	Contig46.RC	Contig46223.RC	Contig49818.RC	Contig51800
Contig55189.RC	Contig55377.RC	Contig56457.RC	Contig753.RC	Contig760.RC
Contig8930.RC	Contig8950.RC	NM.000272	NM.000286	NM.000320
NM.000419	NM.000540	NM.000849	NM.001879	NM.002624
NM.003686	NM.003778	NM.003858	NM.004087	NM.004273
NM.004336	NM.004456	NM.004701	NM.004791	NM.005008
NM.005087	NM.005744	NM.006260	NM.006547	NM.007359
NM.012177	NM.012261	NM.012310	NM.012406	NM.014093
NM.014264	NM.014321	NM.014404	NM.014547	NM.014675
NM.014968	NM.015434	NM.017926	NM.018089	NM.018098
NM.018313	NM.018488	NM.020123	NM.020386	NM.021033

### The validation data set of van de Vijver [4]

A follow-up study by the same group was performed which followed the progression of another set of patients to verify the original study. This follow-up data set contains 295 patients with 106 poor-prognosis and 189 good-prognosis patients. The poor-prognosis patients are categorised into 3 sub-categories.

Patients with metastasis but who did not die as a direct result of the metastasis, patients who died without developing metastasis and the patients who developed metastasis and eventually died. Within these 295 patients, 61 of them are present in the previous study. Those patients will be removed in this paper to ensure we can examine generalisation on a separate set of 234 different patients, 159 of whom are categorised as good-prognosis.

### Topographic Visualisation

Topographic mappings are mechanisms that map the data in a high dimensional space into a low dimensional space in such a way that preserves the structure of the data. This structure of the data usually means the relative distances between points in the high dimensional space, where a suitable distance function is used to reflect the prior knowledge of the domain. In other words, the points that are close together in a high dimensional space should also stay together in a lower dimensional projection space, and data that lie far apart in a high dimensional feature space should remain significantly separated in the lower dimensional projection space.

Both the large quantity of genes and multiple samples of microarray data make it difficult to represent the entire data set and to identify the interesting genes easily. To make understanding easier, a representation is needed to compress all the information in a lower dimensional space. Moreover, each individual patient can be visualised as to whether that patient has expression values closer to the those patients in different prognosis groups. Topographic projection maps do not assume the existence of clusters, boundaries or classes and so are not subject to the same criticisms as supervised approaches for data mining.

In this paper, three reliable topographic techniques are used for embedding the van't Veer data set: NeuroScale [14], Locally Linear Embedding (LLE)and Stochastic Neighbor Embedding (SNE) [17].

### NeuroScale

In a Neuroscale [14] topographic map the distribution and trained relative positions of the points in the projection space are determined to reflect the relative dissimilarity between data measurements (gene expression values) in the high-dimensional space, and hence generalises the established Sammon map concept. *N *measurement vectors **x**_*i *_in ℝ^*p *^are transformed using a Radial Basis Function (RBF) [18,19] network to a corresponding set of feature (visualisation) vectors **y**_*i *_in ℝ^*q*^. An RBF comprises a single hidden layer of *h *neurons which represents a set of basis functions, each of which has a centre located at some point in the input space. Generally, *q *≪ *p *as dimension reduction is desired, and typically *q *= 2 for visualisation. The RBF is a semi-parametric kernel model such that **y**_*i *_= **W Φ **(**r**_**i**_), where the set of weights *W *can be optimised from a training data set. Thin plate spline functions **Φ **(**r**) = **r**^2 ^log(**r**), where $r_{i}^{2}$ = ||**x**_*i *_- **c**|| will be used in this experiment. In the Radial Basis Function network trained traditionally for regression problems, the desired output of the network or the target values are already identified through a supervised learning problem. However, in order to use an RBF in the topographic reduction problem, which does not have predefined targets but only considers the distance-preserving nature of the target values, the traditional supervised Radial Basis Function network needs to be modified by using *relative supervision *where it is only the relative distances between pattern pairs which are important [15]. The quality of the projection is measured by the *Sammon stress metric *(n.b. we are using a reduced form here, neglecting a denominator often employed):

$$E=\sum_{i}^{N}\sum_{j}^{N}{\left(d_{ij}^{*}-d_{ij}\right)}^{2},$$

where *d*_*ij *_= ||**y**_*i *_- **y**_*j*_|| and $d_{ij}^{*}$ = ||**x**_*i *_- **x**_*j*_|| represent the inter-point distances in projection space and data space respectively. The aim of the training process is to set the parameters of the RBF weight matrix to minimise the stress metric and hence capture the the functional relationship between the original data distribution and the projected images. The NeuroScale model is used to visualise and interrogate our data set, considered as subsets of an array of 78 × 70 patterns. Once the functional mapping has been obtained using NeuroScale, the model can be reused without reconstructing the projection over the extended data by just passing the new data points, **x**_*new *_through the transformation function. **y**_*new *_= *f *(**x**_*new*_, **W**).

The number and location of the centres needs to be determined. However choices of centres are robust to the outcome [20] since an implicit smoothing regularisation is used as part of the optimisation process. As a normal practice, the number of centres is chosen to be the same as the number of training data points, so that each data point can be used as a centre of the RBF functions. In this experiment the Netlab toolbox [7] is used to help construct the NeuroScale projections.

### LLE

Locally Linear Embedding (LLE) [16] aims to preserve the local neighbourhood area around a point so that nearby points in high dimensional space remain nearby and similarly co-located with respect to one another in the low dimensional space by preserving the neighbouring distance linearly. Provided there is sufficient data, we expect each data point and its neighbours to lie on or close to a locally linear patch of the manifold. In the simplest formulation of LLE, the method will preserve weights for each data point to the surrounding *K *nearest neighbours per data point, as measured by Euclidean distance from a point of interest. The optimal weights for each data point to the surrounding *K *nearest neighbours are given by:

$$W_{ij}=\frac{\sum_{k}C_{jk}^{i-1}}{\sum_{lm}C_{lm}^{i-1}}.$$

where $C_{jk}^{i}$ is a covariance matrix within the neighbourhood of **x**_*i *_and *η*_*j *_is the neighbour of the data point *x*_*i*_.

$$C_{jk}^{i}={\left(x_{i}-\eta_{j}\right)}^{T}\cdot (x_{i}-\eta_{k}).$$

Each high dimensional point **x**_*i *_is mapped to a low dimensional point **y**_*i *_in low dimensional space, representing global internal coordinates on the manifold. This is done by choosing *d*-dimensional coordinates **y**_*i *_to minimise the cost function in low dimensional space:

$$\Phi (Y)=\sum_{i}^{N}|y_{i}-\sum_{j}^{K}W_{ij}y_{j}{|}^{2},$$

where *W*_*ij *_is fixed from the high dimensional space. This algorithm has only one free parameter: the number of neighbours per data point, *K*. The higher the value of *K*, the more similar to the NeuroScale method this method will be. This *K *practically, is very hard to find to suit the given data set.

Furthermore, it is hard to find an appropriate value of *K *which performs well across different choices of data sets. It is typically much smaller than the number of data points. In the experiments here we will show results using *K *= 5, 20. Furthermore, LLE has a further disadvantage over NeuroScale in that the NeuroScale model can produce a transformation function which can be used for generalisation. Any new patient gene vector can be projected down by using this existing function without recomputing a full new projection, which can be very computational expensive. In addition, the result using LLE is sensitive to the choice of neighbours, *K *while NeuroScale gives quite consistent results [20].

### Stochastic Neighbor Embedding

Stochastic Neighbor Embedding [17] also uses a pairwise similarity but measures similarity using a probabilistic distance approach to preserve the neighbourhood identity. A Gaussian distribution is centred on each object point in the high dimensional space and a probability density is defined over all the potential neighbours of that point. This approach permits a 1-to-many mapping of high dimensional points to projection space.

The high dimensional related probability for each point, *i*, and each potential neighbour, *j*, is computed using the asymmetric probability, *p*_*ij*_, that *i *would pick *j *as its neighbour.

$$p_{ij}=\frac{\exp(-d_{ij}^{2})}{\sum_{k\neq i}\exp(-d_{ik}^{2})}.$$

The dissimilarities, *d*_*ij *_can be based on standard Euclidean distances and scaled by a smoothing factor *σ*_*i *_which is empirically determined:

$$d_{ij}=\frac{\left||x_{i}-x_{j}|\right|}{\sigma_{i}^{2}}.$$

The low dimensional images **y**_*i *_of the points are used to define a probabilistic density in the mapping space, as:

$$q_{ij}=\frac{\exp(-||y_{i}-y_{j}|{|}^{2})}{\sum_{k\neq i}\exp(-||y_{i}-y_{k}|{|}^{2})}.$$

The aim of this SNE method is to match the above two distributions as close as possible. The Kullback-Leibler divergence, which is a measure of dissimilarity between two probabilities is used here as a cost function. This can be achieved by manipulating the coordinates *y*_*i *_to minimise the cost:

$$C=\sum_{i}^{N}\sum_{j}^{N}p_{ij}\log\frac{p_{ij}}{q_{ij}}=\sum_{i}^{N}KL(P_{i}||Q_{i}).$$

The SNE model can be extended for multiple projections of a single object by using a mixture of densities, which produces a probabilistic density in the mapping space:

$$q_{ij}=\sum_{b}\pi_{i_{b}}\sum_{c}\frac{\pi_{j_{c}}\exp(-||y_{i_{b}}-y_{j_{c}}|{|}^{2})}{\sum_{k}\sum_{d}\pi_{k_{d}}\exp(-||y_{i_{b}}-y_{k_{d}}|{|}^{2})}.$$

The number of clusters in the mixture also needs to be determined empirically. Each data point *x*_*i *_can be projected to many locations of the mixtures $y_{i_{b}}$ or $y_{j_{c}}$. However, for the experiments in this paper, only one projection per data will be used. The main advantage of SNE is its probabilistic approach but the results of the SNE are strongly dependent on the chosen *σ*. If the chosen *σ *is too large, the projecting data is likely to collapse to a single point. The suggested *σ *is *σ *= log(*K*), where *K *is the number of neighbours used to define a local cluster. To be consistent with the LLE method, which used *K *= 5, 20, we therefore choose *σ *= log(5), log(20) in the experiments in this paper as representative examples.

### Classifier

Purely for comparison to previous works in this area, we additionally superimpose the results from a discriminative classifier on the projective maps. Classifiers are created to determine the performance of the discrimination patients into good or poor prognosis from each topographic projection images by trying to compare the performance using different techniques and gene lists. The classifiers are built on the two dimensional input of the visualisation space using a separate RBF nonlinear classifier. The output, instead of crisply divided the results into good or poor prognosis patients produces an analogue value indicating the likelihood of good prognosis typically varying from 0 to 1. The classifier uses 2 coordinate input values and produces 2 output values indicating good and poor prognosis likelihood respectively and is trained using the original 78 patients only as a training set. Specifically, the desired target value is **T **= {*T*_1_, *T*_2_} where **T **∈ {[1, 0], [0, 1]} represents good and poor prognosis patients respectively. The two dimensional output of the RBF network is: **y**_*i *_= **W Φ **(**x**_*i*_) where the basis functions constituting **Φ **are selected using cross validation.

The outputs of the RBF network are then transformed using the softmax function, giving a vector prognosis indicator for each patient $P=\frac{\exp(y)}{\sum_{j}\exp(y_{j})}$. One of the two scales outputs which represents the good prognosis class is used as an indicator and contours of the indicator values are superimposed on the projection map space to show the likelihood of the good prognosis indicators.

Patients with predicted prognosis values in the range 0.3 → 0.7 are considered as ambiguously classified. Removing these 'low-confidence' patients from measures of classification performance figures are likely to improve classification rates since the low confidence patients fall in the overlap between the good and poor prognosis groups as will be seen in the latter results.

## Results

In this section we apply three different projective embeddings which are described in Methods, applied to the two PGLs of 70 genes per patient: List A, the van't Veer choice of genes, and List B, an alternative choice of genes chosen for consistency, as described in Methods.

### NeuroScale Projection

**Figure 1:**
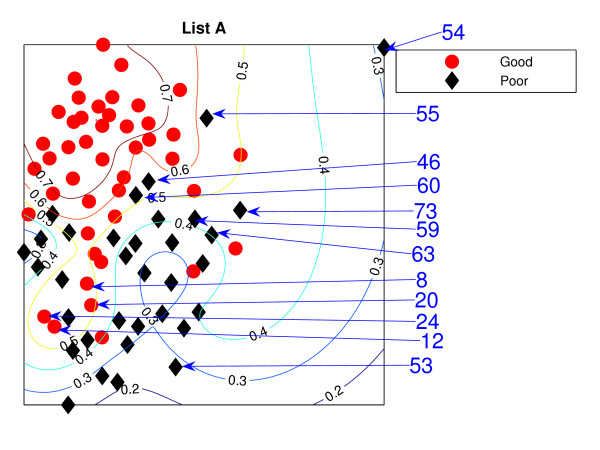
**The NeuroScale results using List A**. NeuroScale map of gene List A. Note the approximate separation of the centroid between poor (diamonds) and good (circles) prognosis groups. Specific individual patients are highlighted with arrows.

**Figure 2:**
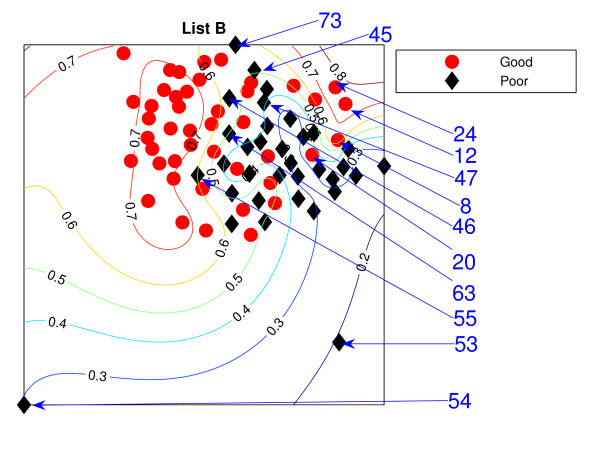
**The NeuroScale results using List B**. NeuroScale map of gene List B. Note the approximate separation of the centroid between poor (diamonds) nd good (circles) prognosis groups. Specific individual patients are highlighted with arrows.

Figure 1 is the result from a 2-dimensional NeuroScale projection using List A and Figure 2 is the result using List B. The group of poor-prognosis and good-prognosis patients are labelled differently, with black diamonds and grey circles respectively. The results similarly show some separation between the two groups of patients with a few patients wrongly mapped into the opposite class. This projection is using each patient data point as a separate centre in the RBF model internal to NeuroScale. However, to emphasize, no class information is used to construct the projection map. The different symbols are simply to allow easier identification of the two patient groups. This projection appears to support the previous result of van't Veer et. al. that List A appears to have some discriminatory capability, although it is evident from these figures that any discrimination is on a graded and overlapping scale rather than providing separable distributions.

Figures 1 and 2, also show the classification model contour lines superimposed on the projection map of NeuroScale. The prognosis indicators vary on the level of overlap between two prognosis groups. The areas where there is large overlap between the two patient groups reflects ambiguity of any likely class membership. Therefore, we regard these patients as low confidence samples as far as determining class information and we regard them as 'unclassifiable'.

Table 1 shows the classification result of the NeuroScale projection using List A with 0.5 prognosis indicator as a threshold boundary between good and poor prognosis signatures of all patients. The overall classification rate is 83.33%, while if only high confidence patients are used for consideration the classification result increases to 100% accuracy, the result shown in Table 2. However, there are only 30 patients that fall in the high confidence regions, less than one half of all patients. Similarly the results using List B, shown in Tables 3 and 4 gave the same classification rate as using the list A but with 4 fewer high confidence patients. The results of the projection maps and the classifications support the previous propositions in the literature regarding the lack of uniqueness of a single PGL.

**Table 1:** The misclassification matrix from the NeuroScale projection using List A. The classification is performed using the original 78 patients with 0.5 prognosis indicator as a threshold boundary. The classification rate is 83.33%.

	Predict Good	Predict Poor
Actual Good	27	7
Actual Poor	6	38

**Table 2:** The misclassification matrix from the NeuroScale projection using List A with only high confidence patients. The classification is performed using only 30 high confidence patients whose indicators are either above 0.7 or below 0.3. The classification rate is 100%.

	Predict Good	Predict Poor
Actual Good	10	0
Actual Poor	0	20

**Table 3:** The misclassification matrix from the NeuroScale projection using List B. The classification is performed using the original 78 patients with 0.5 prognosis indicator as a threshold boundary. The classification rate is 83.33%.

	Predict Good	Predict Poor
Actual Good	28	6
Actual Poor	7	37

**Table 4:** The misclassification matrix from the NeuroScale projection using List B with only high confidence patients. The classification is performed using only 26 high confidence patients whose indicators are either above 0.7 or below 0.3. The classification rate is 100%.

	Predict Good	Predict Poor
Actual Good	7	0
Actual Poor	0	19

### Locally Linear Embedding

**Figure 3:**
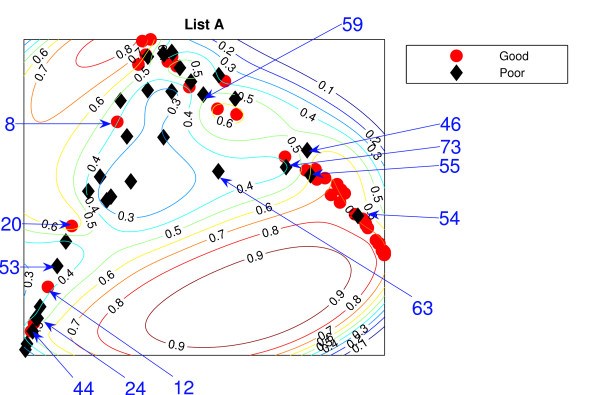
**The LLE results with *K *= 5 using List A**. Projection result of the LLE method using *K *= 5 List A, the van't Veer list: the comparison list. Selected specific patients identified by arrows.

**Figure 4:**
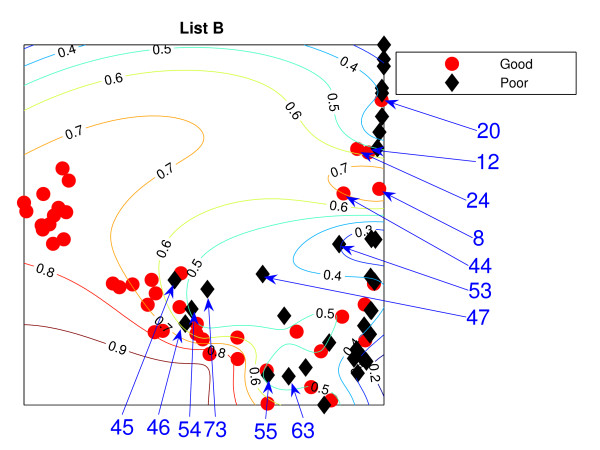
**The LLE results with *K *= 5 using List B**. Projection result of the LLE method using *K *= 5, List B: the comparison list. Selected specific patients identified by arrows.

**Figure 5:**
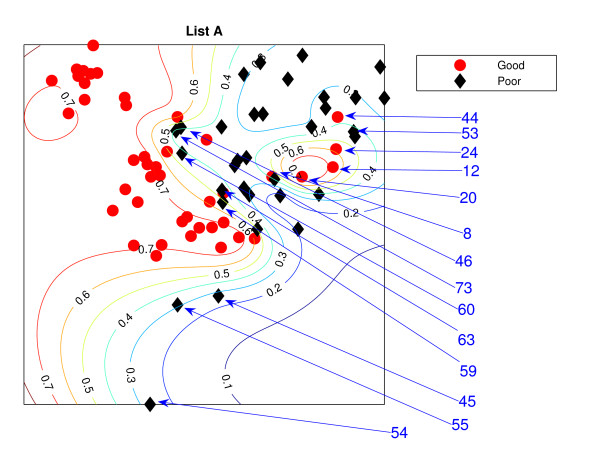
**The LLE results with *K *= 20 using List A**. Projection result of the LLE method using *K *= 20, List A. Selected specific patients identified by arrows.

**Figure 6:**
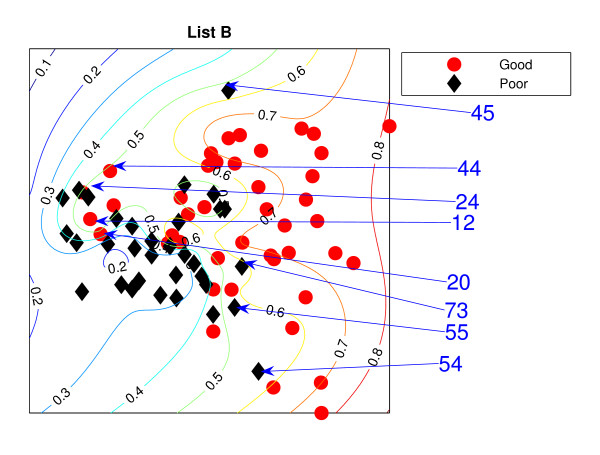
**The LLE results with *K *= 20 using List B**. Projection result of the LLE method using *K *= 20, List B. Selected specific patients identified by arrows.

The Locally Linear Embedding results using two different gene sets are projected down and shown in Figures 3 and 4 using *K *= 5 and Figures 5 and 6 using *K *= 20 together with the classification contour lines of the good prognosis indicator. *K *is the number of neighbours used to construct the mapping which has to be chosen empirically. With *K *= 5, within a good prognosis cluster of both gene lists, there are four obvious poor prognosis patients. Three of them are common across both List projections. These four patients remain in the wrong place even after the number of neighbours increases. Between 13 and 16 poor prognosis patients are likely to be misclassified as good prognosis patients in the 'boundary layer'.

The best representation seems to be *K *= 20 with List A giving slightly better separation with fewer patients misclassified, with 7 good prognosis and 4 poor prognosis patients likely to be misclassified, from inspection of the figures without the classification results. However, some regions can be classified better when the classifier are trained on the particular data set. For example, few good prognosis patients in Figure 5 are on the right of the projection while most of the good prognosis patients are supposed to be on the left side. Those few patients create the region where patients are likely to be good prognosis even though this could be the result of these few outliers.

Both List projections have a separability of the modes of the two groups even though some patients appear in the wrong relative positions for their prognosis groups. Nevertheless, the difficulty for LLE is choosing the appropriate value for *K*. The result shows better separation of the training data with *K *= 20. For Figure 5, poor prognosis patients *P*45, *P*55, *P*54 are isolated from the other patients. However, having these three patients correctly classified could result in poor generalisation across new data. Other than this, the LLE projections reflect some similarities to the NeuroScale projections.

Similar to the NeuroScale classification results, the classification results of LLE are provided in misclassification matrices showing results for all the patients and also using only high confidence patients, with different choices for the number of neighbours, *K*. Tables 5 and 6 show the classification results of LLE with *K *= 5 using List A with classification results on all patients and high confidence patients respectively. Similarly for list B, the classification results for *K *= 5 are shown in Tables 7 and 8. Visually, List B gives a more distinct projection than List A with more clusters of good prognosis patients separated without overlap of many poor prognosis patients. The classification results confirm this. When only high confidence patients are retained, no patients are misclassified using either gene list although the number of high confidence patients using List B is more than using List A by 8 patients.

**Table 5:** The misclassification matrix from the LLE projection using List A with *K *= 5. The classification is performed using the original 78 patients with 0.5 prognosis indicator as a threshold boundary. The classification rate is 79.49%.

	Predict Good	Predict Poor
Actual Good	27	7
Actual Poor	9	35

**Table 6:** The misclassification matrix from the LLE projection using List A with *K *= 5 using only high confidence patients. The classification is performed using only 22 high confidence patients whose indicators are either above 0.7 or below 0.3. The classification rate is 100%.

	Predict Good	Predict Poor
Actual Good	7	0
Actual Poor	0	15

**Table 7:** The misclassification matrix from the LLE projection using List B with *K *= 5. The classification is performed using the original 78 patients with 0.5 prognosis indicator as a threshold boundary. The classification rate is 87.18%.

	Predict Good	Predict Poor
Actual Good	31	7
Actual Poor	3	37

**Table 8:** The misclassification matrix from the LLE projection using List A with *K *= 5 using only high confidence patients. The classification is performed using 29 high confidence patients whose indicators are either above 0.7 or below 0.3. Again a perfect classification rate is achieve.

	Predict Good	Predict Poor
Actual Good	8	0
Actual Poor	0	21

For *K *= 20, the classification results are shown in Tables 9 and 10 for list A and Tables 11 and 12 for list B. Contrary to the *K *= 5 case, the results using List A with *K *= 20 gives better classification performance. The classification rate is quite high with 93.58% accuracy with larger numbers of high confidence patients compared to the other methods, but this could result from the overfitting of this particular model. As can be seen in Figure 5, the gap of the contours between 0.4 to 0.7 is quite narrow. Choosing the exact boundary that determines the prognosis signature of each patient is therefore critical. As a result, if patient values contain uncertain information or noisy data, the resulting classification outcome of such patients is likely to be effectively random. Therefore the data of such uncertain patients should not be taken into account in representing performance results. We investigate generalisation of these results later in the paper.

**Table 9:** The misclassification matrix from the LLE projection using List A with *K *= 20. The classification is performed using the original 78 patients with 0.5 prognosis indicator as a threshold boundary. The classification rate is 93.58%.

	Predict Good	Predict Poor
Actual Good	33	1
Actual Poor	4	40

**Table 10:** The misclassification matrix from the LLE projection using List A with *K *= 20 using only high confidence patients. The classification is performed using 41 high confidence patients whose indicators are either above 0.7 or below 0.3. The classification rate is 100%.

	Predict Good	Piredict Poor
Actual Good	12	0
Actual Poor	0	29

**Table 11:** The misclassification matrix from the LLE projection using List B with *K *= 20. The classification is performed using the original 78 patients with 0.5 prognosis indicator as a threshold boundary. The classification rate is 84.62%.

	Predict Good	Predict Poor
Actual Good	28	7
Actual Poor	5	39

**Table 12:** The misclassification matrix from the LLE projection using List B with *K *= 20 using only high confidence patients. The classification is performed using 28 high confidence patients whose indicators are either above 0.7 or below 0.3. The classification rate is 100%.

	Predict Good	Predict Poor
Actual Good	12	0
Actual Poor	0	16

### Stochastic Neighbor Embedding

**Figure 7:**
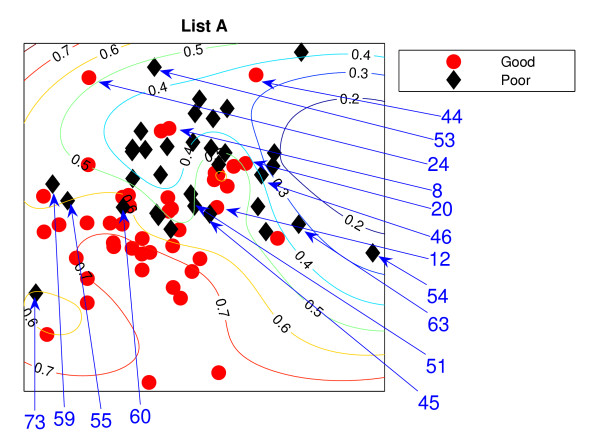
**The SNE results with *σ *= *log*(5) using List A**. The Stochastic Neighbor Embedding projections of List A with *σ *= log(5).

**Figure 8:**
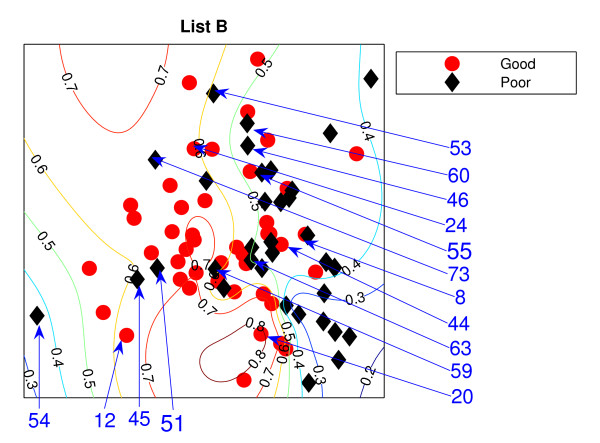
**The SNE results with *σ *= *log*(5) using List B**. The Stochastic Neighbor Embedding projections of List B with *σ *= log(5).

**Figure 9:**
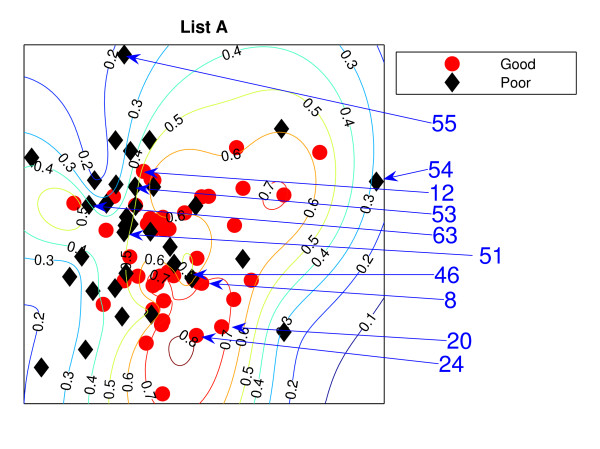
**The SNE results with *σ *= *log*(20) using List A**. The Stochastic Neighbor Embedding projections of List A with *σ *= log(20).

**Figure 10:**
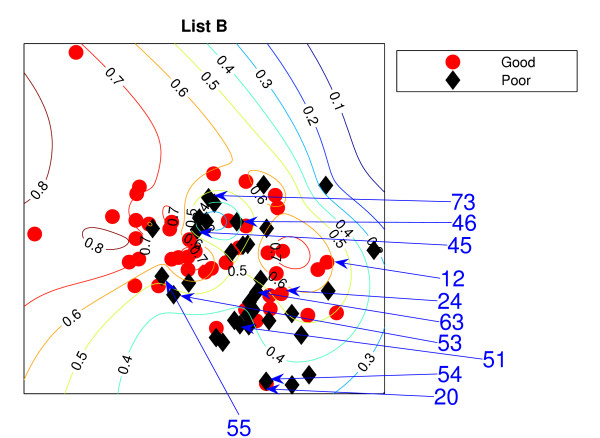
**The SNE results with *σ *= *log*(20) using List B**. The Stochastic Neighbor Embedding projections of List B with *σ *= log(20).

The projection maps of Stochastic Neighbor Embedding reflect different results of qualitative projections for *σ *= log(5) shown in Figures 7 and 8, and *σ *= log(20) shown in Figures 9 and 10. From these figures it can be seen that the relative distributions of the patient projections are quite different for differing choices of the value of *σ *and this value is quite hard to determine. With *σ *= log(20), patients from both gene groups are mostly overlapped. The separation is not as good as in the previous two models.

Figures 7 and 8, show the classification contour lines superimposed on the SNE projection maps using the two different gene Lists with *σ *= log(5), and Figures 9 and 10 with *σ *= log(20).

Tables 13 and 14 show the classification results of the SNE with *σ *= log(5) using List A, with classification results shown on all patients and also those high confidence selected patients respectively. For List B, the classification results are shown in Tables 15 and 16. With *σ *= log(5), List B gives better overall performance but when only high confidence patients are being measured, no patients are misclassified with almost the same number of high confidence patients using both gene lists. Again, this supports the proposition that equivalent performance can be obtained on dissimilar gene lists.

**Table 13:** The misclassification matrix from the SNE projection using List A with *σ *= log(5). The classification is performed using the original 78 patients with 0.5 prognosis indicator as a threshold boundary. The classification rate is 79.49%.

	Predict Good	Predict Poor
Actual Good	27	9
Actual Poor	7	35

**Table 14:** The misclassification matrix from the SNE projection using List A with *σ *= log(5) using only high confidence patients. The classification is performed using 16 high confidence patients whose indicators are either above 0.7 or below 0.3. The classification rate is 100%.

	Predict Good	Predict Poor
Actual Good	4	0
Actual Poor	0	12

**Table 15:** The misclassification matrix from the SNE projection using List B with *σ *= log(5). The classification is performed using the original 78 patients with 0.5 prognosis indicator as a threshold boundary. The classification rate is 80.77%.

	Predict Good	Predict Poor
Actual Good	25	6
Actual Poor	9	38

**Table 16:** The misclassification matrix from the SNE projection using List B with *σ *= log(5) using only high confidence patients. The classification is performed using 17 high confidence patients whose indicators are either above 0.7 or below 0.3. The classification rate is 100%.

	Predict Good	Predict Poor
Actual Good	8	0
Actual Poor	0	9

For *σ *= log(20), the classification results are shown in Tables 17 and 18 for list A, and Tables 19 and 20 for list B. List A gives only slightly better performance to list B which is 79.49% to 74.36%. Both of them gave perfect classification rates when restricted to high confidence patients although list B has more high confidence patients (14 patients), compared to 10 patients in list A. Nevertheless, the number of retained high confidence patients in this method is very low.

**Table 17:** The misclassification matrix from the SNE projection using List A with *σ *= log(20). The classification is performed using the original 78 patients with 0.5 prognosis indicator as a threshold boundary. The classification rate is 79.49%.

	Predict Good	Predict Poor
Actual Good	24	10
Actual Poor	6	38

**Table 18:** The misclassification matrix from the SNE projection using List A with *σ *= log(20) using only high confidence patients. The classification is performed using 10 high confidence patients whose indicators are either above 0.7 or below 0.3. The classification rate is 100%.

	Predict Good	Predict Poor
Actual Good	2	0
Actual Poor	0	8

**Table 19:** The misclassification matrix from the SNE projection using List B with *σ *= log(20). The classification is performed using the original 78 patients with 0.5 prognosis indicator as a threshold boundary. The classification rate is 74.36%.

	Predict Good	Predict Poor
Actual Good	25	11
Actual Poor	9	33

**Table 20:** The misclassification matrix from the SNE projection using List B with *σ *= log(20) using only high confidence patients. The classification is performed using 14 high confidence patients whose indicators are either above 0.7 or below 0.3. The classification rate is 100%.

	Predict Good	Predict Poor
Actual Good	2	0
Actual Poor	0	12

## Discussion

### Comparison across models

The three methods gave different visualisation outcomes. LLE with *K *= 5 represents the data as a quasi 1-dimensional representation while the other two models present 2-dimensional mappings. However on inspection, they reveal some similarities. For both gene sets, poor prognosis patients whose gene feature vectors are significantly placed in the wrong cluster are consistently misplaced across models. For LLE, there are 4 poor prognosis patients (in both gene sets) who are projected to the wrong cluster. Recall that the feature sets used are almost non-overlapping. These patients may be used to compare between models. For List A, instead of *P*73 using LLE, *P*60 is having a low confidence and more likely to be misclassified. Only NeuroScale places *P*54 far from the remaining patients. We note that *P*54 is exceptional in that the patient's gene list has several missing values (and for this reason was eliminated from analysis in the paper by Ein-Dor [1]). The NeuroScale model correctly identifies *P*54 as an outlier patient requiring further investigation. Note that a classification model built from this projection would place *P*54 into a good or poor prognosis class despite the missing information.

With *K *= 20 in LLE, three patients, *P*54, *P*55, *P*45, are separated from the remaining patients, instead of clustering amongst the other good-prognosis patients as indicated by the other projection models. With this number of neighbours in LLE, the projection is giving better patient group separability and giving correct classification results despite being outliers.

However, for the SNE projective visualisation, *P*54 has a surprisingly high confidence of being correctly classified and does not reflect the problems of missing information. Instead of patient *P*54, *P*59 is misclassified by the SNE projection but with low confidence. On the other hand the likely misclassified good prognosis patients are common using both LLE and NeuroScale but with some slight difference to the SNE is that *P*12 does not significantly project to the wrong cluster.

In addition, for List B, both LLE and NeuroScale give similarly consistent results for projections of good and poor prognosis patients into the incorrect groups as shown in both visualisation and classification results. The difference using SNE is that SNE gives a better representation of *P*46 but gives an incorrect projection to *P*51 instead. Similarly, the significantly misclassified good prognosis patients are the same using both LLE and NeuroScale but it is very difficult to discriminate using Stochastic Neighbor Embedding.

Both LLE and SNE show sensitivity of projections to empirical choices of selectable parameters, but projections can be found some consistency of patient distribution across all three nonlinear topographic projection methods. However, NeuroScale has an advantage over the other two methods because of its principled basis on a machine learning parameterised mapping that can be reused in a generalisation experiment without the need to retrain any models. We will test this feature shortly.

### Comparison of PGLs across patient groups

Both gene lists A and B produce similar projections, except that *P*55 in List A is mostly projected to the wrong group by all three models. However, with List B it is not as unambiguous since this patient is projected into the interface between the two prognosis groups. *P*45 appears as one of the wrongly projected poor prognosis patients instead of *P*55. Except for these two patients with different results, both gene lists create similar projections, despite the fact that both gene sets have very few genes in common. This supports the opposing view, that different gene lists can be created from small sample patient groups which randomly correlate with arbitrary outcome. The classification results confirm the similarity using both gene lists.

From the observations, most patients have similar representations in the projected mappings. Some patients are better represented by one PGL or the other. Nevertheless both gene lists produce an overlapping patient region in which patients in this area cannot be separated into either good or poor prognosis groups. The clinical prognosis of these patients should be *unclassifiable *instead of being assigned into any one prognosis group. This new patient type can be crucial in the medical domain where the advice to the clinician should be that no prediction can be made on the available information and extra information is needed in addition to the gene expression profile.

### Generalisation results using the van de Vijver data set

In the earlier studies of van't Veer et. al., validation of the original 70 PGL was made by obtaining good performance on an additional patient data set. In this section we perform the same comparison, making use of the functional mapping ability of NeuroScale applied to the extended van de Vijver data.

NeuroScale has an advantage over some other topographic models in that new data can be projected through a prior learned projection mapping. Once the functional mapping has been obtained using NeuroScale, the model can be reused without reconstructing the projection using novel data. For additional comparison, this section will also use a retrained LLE model (trained on the new full data set) with *K *= 20 which performed well with the previous patient set, as a comparison to the generalised NeuroScale. For validation, the new patient set of van de Vijver will be projected using the same networks for both gene lists. In their study [4], they verified the viability of their 70 nominated genes as prognosis indicators of breast cancer by using the previous 78 patients as a training set and testing on this new patient set. We will investigate this claim further by applying trained topographic visualisation to this new patient set using both PGL lists A and B, and observe the consistency of the distribution between the two groups of patients.

**Figure 11:**
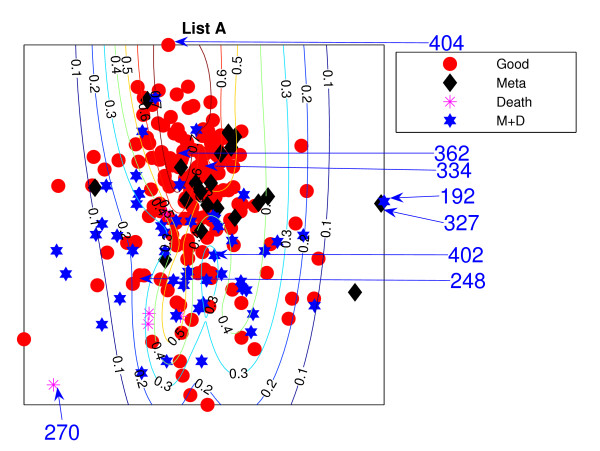
**The van de Vijver NeuroScale projection map with List A**. The NeuroScale Visualisation projection of the new 234 patients trained using the original 78 patients based on List A. Circles represent healthy patients who did not develop any further sign of relapse, asterisks for patients who developed metastasis but did not die, diamonds for the patients who died without developing metastasising cancer and stars for patients who developed metastasis and then died consequently.

**Figure 12:**
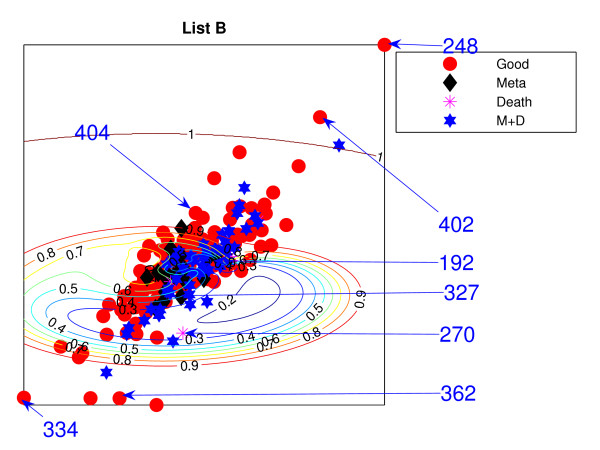
**The van de Vijver NeuroScale projections with List B**. The NeuroScale Visualisation projection of the new 234 patients trained using the 78 patients based on List B. Circles represent healthy patients who did not develop any further sign of relapse, asterisks for patients who developed metastasis but did not die, diamonds for the patients who died without developing metastasising cancer and stars for patients who developed metastasis and then died consequently.

Figures 11 and 12 show the projections of those remaining 234 patients, labelled into 4 different groups, which are (1) good-prognosis patients (circles), (2) metastasis patients (asterisks), (3) death (stars) and (4) both metastasis and death (diamonds). Both PGL projective visualisations seem to give similar projections on the new data. For List A from Figure 1, good prognosis patients are likely to be projected towards the top of the visualisation map while the poor prognosis patients are at the bottom. The projection of new data for List A in Figure 11 shows some density of good-prognosis patients on the top of the visualisation map, similar to Figure 1. However, many good-prognosis patients are distributed across the visualisation map. For List B, the patient gene vectors are quite dense in Figure 12; however a number of good-prognosis patients tend to be on the top left of the plot, similar to Figure 2.

The results using the two different gene lists are different. Many poor prognosis patients are more clustered in the middle while the good prognosis patients are likely to be more widely distributed. This is especially true for List B. Poor-prognosis patients of list B, visually, are more likely to be separable than using List A. Some patients give different projections: *P*192 and *P*327 are at the right edge of the visualisation projection using List A which give very high confidence of being poor prognosis while List B projects them down into the central regions which give lower confidence of being poor prognosis patients. Alternatively many good-prognosis patients are projected to the central regions using List A but are scattered around the edges using list B: for example *P*362, *P*248 which give very high confidence of being good prognosis patients while list A does not give such high confidence of *P*362 and in addition, misclassifies *P*248.

However, the overall separation between the two patient groups can be seen to be significantly worse for this set of generalisation patients when compared to the original patient set used in the training phase. The classification results of the RBF classifiers trained on the previous 78 patients can be reused with the new 234 patients in the NeuroScale projection space because of its reusability. Tables 21 and 22 show the classification results using List A and Tables 23 and 24 show the classification results using List B. It can be seen that the classification results drop from the previous data set dramatically from around 80% to less than 60% for all patients.

**Table 21:** The misclassification matrix from the NeuroScale projection on the new 234 patients using List A. The classification is performed using the new 234 patients with 0.5 prognosis indicator as a threshold boundary 70 gene set provided by List A. The overall classification rate is 58.97%

	Predict Good	Predict Poor
Actual Good	56	77
Actual Poor	19	82

**Table 22:** The misclassification matrix from the NeuroScale projection on the new 234 patients using List A with only high confidence patients. The classification is performed using only the 78 high confidence from the new 234 patients whose indicators are either above 0.7 or below 0.3 using 70 gene set provided by List A. The overall classification rate improves to 60.26%

	Predict Good	Predict Poor
Actual Good	24	28
Actual Poor	3	33

**Table 23:** The misclassification matrix from the NeuroScale projection on the new 234 patients using List B. The classification is performed using the new 234 patients with 0.5 prognosis indicator as a threshold boundary 70 gene set provided by List A. The overall classification rate is 56.40%

	Predict Good	Predict Poor
Actual Good	32	43
Actual Poor	59	100

**Table 24:** The misclassification matrix from the NeuroScale projection on the new 234 patients using List B with only high confidence patients. The classification is performed using only the 92 high confidence from the new 234 patients whose indicators are either above 0.7 or below 0.3 using 70 gene set provided by List A. The overall classification rate improves to 67.40%

	Predict Good	Predict Poor
Actual Good	13	21
Actual Poor	9	49

Slight improvements in classification are made when only the high confidence patients are retained, increasing performance to slightly more than 60%.

**Figure 13:**
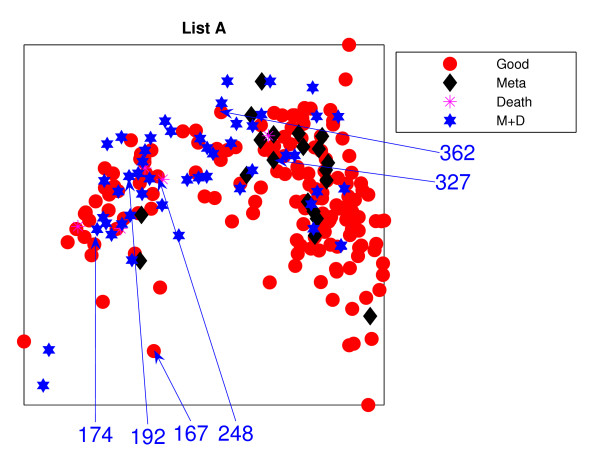
**The van de Vijver LLE projections using List A**. The LLE Visualisation projection of the new 234 patients, excluding the previous 61 patients, trained on the van't Veer study of 78 patients using the 70 gene set of List A with *K *= 20.

**Figure 14:**
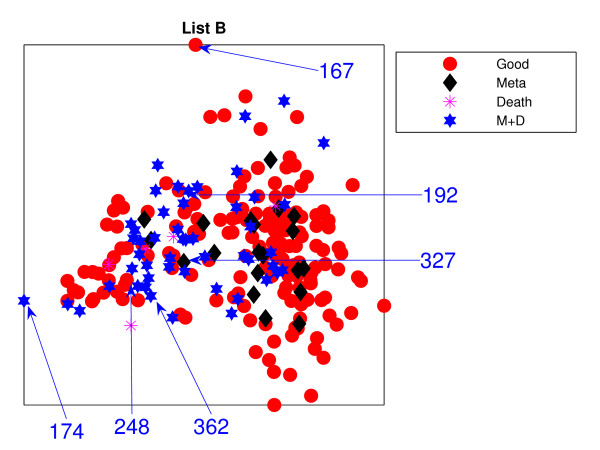
**The van de Vijver LLE projection using List B**. The LLE Visualisation projection of the new 234 patients, excluding the previous 61 patients, trained on the van't Veer study of 78 patients using the 70 gene set of List B with *K *= 20.

For comparison, LLE was *retrained *on the full set of 234 patients and also projected down. The results are shown in Figures 13 and 14 using *K *= 20. These new projections of good or poor prognosis patient groupings have little resemblance to the previous results obtained on the original training set.

There is no separation between the two prognosis groups using the LLE model, which shows the poor consistency between the chosen *K *for the previous patient set and the new patient set. The pre-trained network of the NeuroScale, provides a more separable projection map. Nevertheless, both visualisation models create large sections of overlap between the two patient groups.

For LLE, the classifiers trained from the previous data set can not apply here since the new projections have to be retrained completely using the new data set. Therefore, the classifiers are not suitable for this model. Only the visualisation results are shown.

These results indicate that the selected 70 genes are not representative of robust Predictive Gene Lists for prognosis on this problem domain for these patients. The 70 genes extracted from the original data set performs well only on particular patients due to the random correlation effect occurring because of the large dimensionality of the original 25, 000 genes and the small patient sample size. The two different lists of 70 genes perform equivalently in the projection mapping showing the non-uniqueness of the selected gene list. Only parts of the map may be used to identify the good-prognosis patient cluster with any degree of confidence. However, the broad overlap demonstrated in this paper indicates that most patients should be considered as *unclassifiable *based on these subset PGLs.

In the van de Vijver study, the generalisation performance from the van't Veer data set is mentioned. However, the publication only implemented a Kaplan-Meier analysis which shows the random outcome of the patients predicted as poor prognosis signatures but with a good rate of outcome of patients predicted as good prognosis patients. However, this patient list also contains most of the original 78 patients also, which obviously make the classification rates higher than expected for a genuine generalisation performance test. Furthermore, a definite threshold that divides the good and poor prognosis signatures has been specified. Therefore, patients who are intrinsically unclassifiable are also included in the classification results which is not to be advised.

## Conclusion

We have proposed an alternative approach to investigate patient-specific gene-based breast cancer prognosis by providing a topographic projection mapping approach. A comparison using different non-linear projection models has been demonstrated using a single data set projected by using two almost orthogonal lists of 70 genes as feature vectors for each patient.

However, the separation between two prognosis groups dropped dramatically when the visualisation is applied to a new set of patients. We found that the gene list giving reasonable separability of patient types in the preliminary experiments does not separate patient groups of the later study. In addition, the overlap between patient groups is large and can lead to misleading prognoses, which indicates that using a small number of patient samples to identify gene markers generically yields unreliable results.

Furthermore, both gene lists give similar separability results despite there being only a small overlap of genes between the two feature vectors. However, there are some specific patients whose results are notably distinct between the two PGLs. It can be interpreted that there are more important structures that are hidden across multiple genes which could be related to the development of cancer metastasis. Using patients whose gene expression profiles are unsure of giving a definite class label is ill-advised. These patients have intrinsic uncertainty attached to their gene expression profiles and should not be used in prospective studies without further investigation. An overoptimistic impression of the results is likely to be made if uncertainty issues in the data are ignored.

We conclude that patient-specific prognosis results cannot be determined by looking at only gene expression, especially with only a small subset extracted from large numbers of genes from small patient samples. Many genes are randomly correlated with the survival over a small population sample. Although using gene expression profiles can be one tool which helps toward indicating the outcome of breast cancer, the predictive uncertainty given to the prognosis results is high. In addition, methods should not attempt to provide classification estimators in medical decision support, especially for the group of 'unclassifiable' patients, without indicating the extreme uncertainty attached to each patient. More work is needed on quantifying the patient-specific confidence in prognosis estimators, regardless of the approach.

## Competing interests

The author(s) declare that they have no competing interests.

## Authors' contributions

DL conceived and developed the research plan. MS implemented and developed the algorithms based on the NetLab toolbox and performed the computations. Both authors contributed to writing the manuscript and have both read and approved the final version.

## Pre-publication history

The pre-publication history for this paper can be accessed here:


